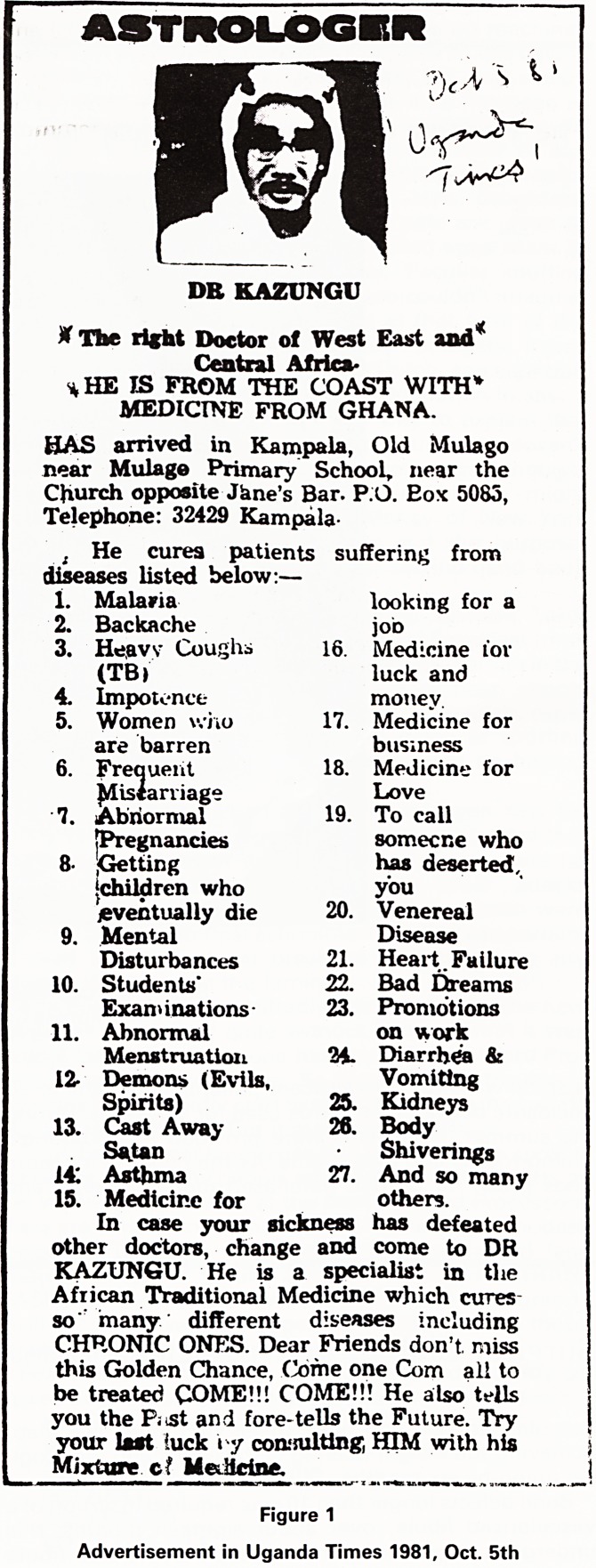# From Our Foreign Correspondent

**Published:** 1986-06

**Authors:** 


### From our Foreign Correspondent

### The Resurgence of the The Witch Doctor in Post-Colonial Africa

**Edward Steen**

Is 'traditional medicine' mumbo-jumbo, as I was given to understand as a doctor's son in then British-run Uganda?

Any link the witch-doctors had with modern times was the constant pinching of syringes from Mulago Hospital. They plunged them into their customers with unsterilised needles, I remember overhearing. Victims often ended up being treated in Mulago, occassionally with cattle urine in their blood.

The natives, don't y' know, had got it into their heads that the syringe was just another kind of magic.

It is, when it is clean. That seems to be the new wisdom after a decade of growth in traditional remedies in newly-independent Africa. The increasingly numerous apologists for traditional medicine, supported by a sizeable faction of the WHO, make much of a 'heightened placebo effect'. They go further (reflecting perhaps a general disenchantment in the West with white-coated medical technicians).

'Around 90–95 per cent of infections are self-healing', says Dr Carol MacCormack, of the London School of Hygiene and Tropical Medicine. 'So you either die or get better. Mostly you get better. Almost anything might appear to help. About 30 per cent of Western medicine is the placebo effect...'

This leading expert in the field started a recent paper with the words: 'Magic is a system of explanation; a way of thinking which seeks to control the environment and achieve desired outcomes'. It was, she argued, unlike science only insofar as its results could not be empirically tested.

Whether one accepts this or not, traditional medicine is the de facto health service of the Third World, above all in country districts – so for 80 per cent of Zimbabwe's 7.5 m people, for instance. There is little money to replace the *n'angas*. Anyway, they are popular, if a little shamefacedly, even in the big cities.

In a chapter of 'The Professionalisation of African Medicine' to be published soon by Manchester University Press, Dr MacCormack mentions a survey of the tiny, élite class of Pakistani women university graduates. Some 40 per cent of them gave birth attended by a traditional midwife.

After slight probing, a sophisticated Zimbabwean diplomat in London, Godfrey Chanetsa, admitted to me that two of his close relatives, one of them his mother, had been convincingly cured by *n'angas* after conventional doctors had despaired. 'Of course the *n'angas* use all sorts of trickery', he said. Indeed both cases seemed to involve essentially psychological troubles, a special forte of traditional healers. They are said to be effective in cases of addiction and skin disorders. Mr Chanetsa also mentioned that *n'angas* were inclined to use a technique in infertility cases which is simple, sometimes highly effective, and the BMA wouldn't approve...

Which raises the question of whether, as is declared policy in many countries, it is possible to meld traditional with modern services. Traditional midwives and bone-setters are often excellent, and would be much better if

**ASTROLOGER**

Oct 5 81 'Uganda Times'

**DR KAZUNGU**

*** The right Doctor of West East and* Central Africa.**
*** HE IS FROM THE COAST WITH* MEDICINE FROM GHANA.**

**HAS arrived in Kampala, Old Mulago near Mulago Primary School, near the Church opposite Jane's Bar. P.O. Box 5085, Telephone: 32429 Kampala.**

**He cures patients suffering from diseases listed below:—**

1. Malaria

2. Backache

3. Heavy Coughs (TB)

4. Impotence

5. Women who are barren

6. Frequent Miscarriage

7. Abnormal Pregnancies

8. Getting children who eventually die

9. Mental Disturbances

10. Students' Examinations

11. Abnormal Menstruation

12. Demons (Evils, Spirits)

13. Cast Away Satan

14. Asthma

15. Medicine for looking for a job

16. Medicine for luck and money

17. Medicine for business

18. Medicine for Love

19. To call somecne who has deserted you

20. Venereal Disease

21. Heart Failure

22. Bad Dreams

23. Promotions on work

24. Diarrhea & Vomiting

25. Kidneys

26. Body Shiverings

27. And so many others.

**In case your sickness has defeated other doctors, change and come to DR KAZUNGU. He is a specialist in the African Traditional Medicine which cures so many different diseases including CHRONIC ONES. Dear Friends don't miss this Golden Chance, Come one Com all to be treated COME!!! COME!!! He also tells you the Past and fore-tells the Future. Try your last luck by consulting HIM with his Mixture of Medicine.**

**Figure 1**

**Advertisement in Uganda Times 1981, Oct. 5th**

Edward Steen is a correspondent to the Sunday Telegraph. His interest in Africa began as a child when his father, Dr Terence Steen, the Bristol Anaesthetist, was working at Mulago Hospital, Kampala, Uganda in the perhaps now late-lamented colonial era. -Ed. *(See page 66)*

taught the rudiments of hygiene: infected wounds and neo-natal tetanus are common problems. But Dr MacCormack is sceptical about the chances or the wisdom of imposing standards and building professional associations: – 'We know the side-effects of that in the West'.

In any event, *n'angas* were not prepared to play ill-paid second fiddle as auxiliaries, she thought. Their legitimacy consisted in a mystical or inherited wisdom, holiness, and could not be separated from their herbal and other skills. The 'professional body' which quite effectively weeds out incompetents or charlatans, argues Pamela Reynolds, another contributor to the book mentioned above, is the common sense of the local community.

I wonder (out of filial loyalty only?)

There can be ticklish problems, flowing from the ingrained belief that no-one dies, or even suffers a misfortune, without someone else, or a spirit, willing it. In the cosmology the Christians could not extirpate, there is a *reason* for everything beyond, for instance, not having looked before you stepped into the road and were hit by a bus.

A benign *n'anga* might use the opportunity of this accident to help his patient set his mental and social house in order – like being kinder to his mother, more respectful to his father's memory – as well as setting his bones. But opportunist healers trying to carve out new territory can be a devilish nuisance.

From the *Zimbabwe Herald*, spring 1985: 'Last week we published the sad story of a teacher, Cde Mixon Nyamunetsa, who was accused of bewitching his uncle to death and was brutally beaten and robbed of seven head of cattle to pay for his alleged crime. It would seem that the belief in witchcraft, sniffing of witches and public accusations of witchcraft has increased tremendously since independence, as court cases reveal. It suggests that the advent of independence and African rule is mistaken by superstitious elements and exploiters of African primitive beliefs for monetary gain, for a return to the antiquarian Stone Age period. This is aided and abetted by official silence on the issue ... if not active support.'

It is true that Zimbabwe has been especially keen on traditional medicine, in part because it, the bête noire of the colonists, can be used to forge a sense of African identity before the whites arrived. 'It gives a sense of history', says Mr Chanetsa.

The excitable former Health Minister Dr (med) Herbert Ushewokunze was behind the headlong growth of ZINATA, the 10,000-strong Zimbabwe National African Traditional Association. Dr Ushewokunze, who named all his seven sons Herbert in his own honour, is Transport Minister at the time of writing. ZINATA lives on. Sceptics might ask about the attendant problems:is it enough to wait until the 'local community' – for me always a suspect expression – turns the tables on the mountebanks by accusing them, in turn, of being witches too?

It is remarkable how long loopy people of a certain type can hold their fellows in thrall. On my last visit to Uganda, in October 1981, I cut out the advertisement of Dr Kazungu, 'astrologer' (Fig. 1) who had 'arrived from the coast with medicine from Ghana'. His list of cures had 27 items, going from malaria, 'Cast Away Satan' at no. 13, medicine for looking for job, venereal disease, kidneys, body shiverings, and the last, 27, 'And so many others'. What does one make of Dr Kazungu?

'An exotic' said Dr MacCormack dismissively, as the buses rumbled by outside the School of Hygiene in Bayswater, each one charged, perhaps, with destiny and meaning.

## Figures and Tables

**Figure 1 f1:**